# Mechanism of lysine oxidase-like 1 promoting synovial inflammation mediating rheumatoid arthritis development

**DOI:** 10.18632/aging.205429

**Published:** 2024-01-12

**Authors:** Jiawei Hu, Xuqiang Liu, Qiang Xu, Meisong Zhu, Song Wang, Kun Quan, Min Dai, Fengbo Mo, Haibo Zhan

**Affiliations:** 1 Department of Orthopedics, The 1st Affiliated Hospital, Jiangxi Medical College, Nanchang University, Nanchang, Jiangxi Province 330006, China

**Keywords:** lysine oxidase-like 1, rheumatoid arthritis, PI3K/AKT, basement membranes, synovitis

## Abstract

Rheumatoid arthritis (RA) is a chronic inflammatory joint disease that causes great distress to patients and society. Early diagnosis is the key to the successful treatment of RA. The basement membrane, one of the oldest tissue structures, is localized under the epithelium. Its complex composition and rich biological functions have made it a focus of research in recent years, while basement membrane-associated genetic variants are involved in most human disease processes. The aim of this study is to find new diagnostic biomarkers for RA and explore their role and possible mechanism in rheumatoid arthritis. The GSE12021, GSE55235 and GSE55457 datasets were downloaded from the GEO database. Their fraction associated with basement membrane genes was analyzed and differentially expressed genes between the disease and normal groups were explored. We identified two basement membrane-associated genes, lysine oxidase-like 1 (LOXL1) and discoid peptide receptor 2 (DDR2). Focusing on the more interesting LOXL1, we found that LOXL1 expression was significantly elevated in the synovium of patients with rheumatoid arthritis, and LOXL1 mRNA and protein levels were elevated in tumor necrosis factor α-stimulated human synovial sarcoma cells (SW982). And LOXL1 knockdown inhibited tumor necrosis factor α-induced inhibition in SW982 cells expression of inducible nitric oxide synthase (INOS), cyclooxygenase-2 (COX2), and interleukin-6 (IL-6). Interestingly, knockdown of LOXL1 inhibited the phosphorylation of PI3K and AKT. In summary, LOXL1 may become a novel diagnostic gene for RA, and knockdown of LoxL1 may inhibit synovial inflammation by affecting PI3K/AKT pathway.

## INTRODUCTION

Rheumatoid arthritis is a common chronic autoimmune joint disease characterized by progressive joint destruction, damage to various organs throughout the body, and a high disability rate [1]. In recent years, rheumatoid arthritis has shown an ever-growing prevalence and incidence, especially in developed countries or regions. Notably, this trend is more pronounced in females. Despite the increasing prevalence of RA, the severity of the disease, end-stage patient mortality, and other systemic complications appear to be decreasing [2, 3]. Unfortunately, mortality in patients with rheumatoid arthritis remains higher than in healthy individuals, and the treatment of cardiovascular and other systemic complications remains a great challenge [4]. As RA has been studied in recent years, a large number of biologic and small molecule kinase inhibitors have been introduced. However, they may still ultimately exert their roles by affecting downstream tumor necrosis factor and interleukin 6 [5]. For now, early diagnosis of RA remains the key to achieve optimal therapeutic outcomes, which makes it interesting to explore new diagnostic biomarkers for rheumatoid arthritis and analyze the link between their key genes and phenotypes.

Basement membranes (BMs) are extracellular matrixes widely distributed in tissues, with core components composed of laminin, type IV collagen, glycoprotein nidogen, acetyl heparan sulfate proteoglycans and some specific proteins. They are localized beneath epithelial cells and not only play a structural support role in biological tissues, but also play an important role in cellular information interaction, blood filtration, tissue fibrosis process, vascular and tumor cell growth, etc., [6, 7]. The pathological alterations of basement membranes play a role in the progression of common diseases such as myocardial infarction and diabetic retinopathy on the one hand, and for many autoimmune diseases, the constituents of the basement membrane are involved in the development of many diseases as target antigens for immune recognition attacks on the other [8–10]. Mutations in more than 20 genes encoding basement membrane proteins and basement membrane-associated proteins underlie multiple diseases affecting each major organ system, further highlighting the central role of basement membranes in human diseases and their diversity [11]. A recent bioinformatic analysis-based study of basement membrane genes defined an integrated network of 224 human proteins and their animal homologs located in the basement membrane, providing new insights into basement membrane function and its impact on diseases [12]. With recent innovations in data processing technology, high-throughput gene microarray analysis of normal subjects and patients enables us to understand the disease at multiple levels, from somatic mutations to genomic expression at the transcriptional level, proteomics and epigenetic alterations, helping us to more comprehensively understand the disease process of rheumatoid arthritis [13–15]. As a typical autoimmune disease, rheumatoid arthritis affects the function of major organs throughout the body as the disease progresses. Although considerable progress has been made in understanding the pathogenesis of rheumatoid arthritis, studies on the role of basement membrane-associated genes in the development of rheumatoid arthritis have not yet been reported.

Lysyl oxidase (LOX) is a copper-dependent monoamine oxidase. In mammals, the lysyl oxidase family consists of five members, namely, lysyl oxidase (LOX) and four lysyl oxidase-like proteins (LOXL1-4). Members of this enzyme family not only contain a copper ion-binding region, but also tyrosine and lysine residues in their conserved C-terminal structural domains, forming the lysyl-tyrosine-quinone cofactor. These common structures lead to similar amine oxidase activities of their family members, oxidizin primary amine substrates to reactive aldehydes [16]. LOXL1, LOXL2 and LOXL4 have been determined to be stably expressed on basolateral membranes in humans and vertebrates [12]. Conventional wisdom holds that the lysyl oxidase family functions to catalyze the formation of lysine-derived cross-links in fibrillar collagen and elastin [17]. Lysine oxidase-like protein 1 (LOXL1), a member of the lysyl oxidase family, is involved in elastin biosynthesis and collagen cross-linking, as well as in the development of injury, fibrosis and cancer. Aberrant expression of LOXL1 contributes to the development of several diseases due to the diversity of lysyl oxidase-like protein 1 functions. Ma et al. showed that the expression of LOXL1 was increased in fibrotic liver diseases, and the knockdown of LOXL1 could inhibit transforming growth factor-β1-mediated proliferation and fibrosis of hepatic stellate cells by affecting the phosphorylation of Smad2/3 [18]. While investigating the formation of fibrotic foci associated with inflammation in breast cancer, Jeong et al. found that LOXL1 expression was not only closely associated with intratumoral inflammation, but also significantly correlated with interleukin-4 [19]. However, the expression and regulatory mechanisms of LOXL1 in RA remain unclear.

In this study, we used bioinformatics and machine learning to find new basement membrane-associated genes for rheumatoid arthritis diagnosis and to explore their role in disease progression. First, we analyzed the association between the basement membrane-associated gene network and three GEO datasets to identify differentially expressed genes between RA patients and healthy specimens. Then, we determined their diagnostic value and explored their immune mechanisms based on machine learning in office automation. Next, we clarified our conjecture by collecting patient’s specimens for validation and *in vitro* gene expression validation. Finally, we proposed and validated that the differentially expressed gene LOXL1 may influence the disease process by affecting the PI3K/AKT signaling pathway mediating inflammation in rheumatoid arthritis synovial cells.

## MATERIALS AND METHODS

### Clinical samples

In the experimental group, synovial tissue was obtained from 6 patients with chronic osteoarthritis, including 2 males and 4 females, aged 48–87 years. Synovial tissues in the control group were obtained from patients with violent knee ligament or meniscus injury requiring knee arthroscopic surgical intervention to clear proliferated synovium, with no history of osteoarthritis or rheumatoid arthritis, in 3 males, aged 25–32 years. The population demographic information is summarized in Table 1. All human genetic resources involved in the experiment were used with the written informed consent of the patients. The study was approved by the Ethics Committee of the First Affiliated Hospital of Nanchang University.

**Table 1:** Population demographic information of clinical samples.

**Sample type**	**Samples**	**Average age**	**Race**	**Gender**		**Sampling site**
				**Female**	**Male**	
Control	3	27.33	Asian	0	3	knee joint
Osteoarthritis	3	75.67	Asian	2	1	knee joint
Rheumatoid arthritis	3	62.33	Asian	2	1	knee joint

### Cell culture

The human synovial cell line SW982 was purchased from Pricella (Wuhan, China). The cells were cultured in DMEM containing 10% extra fetal bovine serum, 100 U/ml penicillin and 100 μg/ml streptomycin at 37°C with 5% CO2 in air. The culture medium was changed once every 2–3 days, and the cells were passaged when the cell fusion reached 90% or more. Tumor necrosis factor-α (TNF-α) used for *in vitro* stimulation was purchased from Proteintech (Wuhan, China). The cells were stimulated with a certain concentration of TNF-α for a period of time and collected for subsequent experiments.

### Real-time quantitative PCR (RT-QPCR)

Total RNA was extracted from cells or tissues using TRIZOL reagent (Thermo Fisher Scientific, USA). RNA (1 μg) was reverse transcribed into complementary DNA using reverse transcriptase (Takara Bio, Shiga, Japan) according to the manufacturer’s protocol. The A260/A280 ratio was calculated to verify the purity and quality of cDNA. RT-PCR was performed using CFX Connect (Bio-Rad, Shanghai, China) with TB Green Premix Ex Taq II (Takara Bio) by denaturing at 95°C for 5 s and annealing at 60°C for 40 s for 42 cycles. Gene expression was analyzed by the 2-ΔΔCt method using glyceraldehyde-3-phosphate dehydrogenase (GAPDH) levels as an internal control. Each gene analysis was repeated at least three times. The primer sequence is shown in Supplementary Table 1.

### Tissue and cellular protein extraction and western blotting experiments

Proteins were extracted from frozen human synovial tissue or cultured SW982 cells into cold RIPA buffer containing protein hydrolase inhibitor and phosphatase inhibitor. Protein samples were taken after centrifugation, then separated by SDS-PAGE and transferred to PVDF membranes (Thermo Fisher Scientific). After sealing with 5% skim milk for 2 h, primary antibody was incubated overnight. The membranes were then washed three times with TBST, followed by co-incubation with horseradish peroxidase (HRP)-coupled secondary antibody for 2 h. Finally, the membranes were visualized by enhanced chemiluminescence, with GAPDH as a control. The grayscale values of the bands were analyzed by ImageJ software and statistical analysis was carried out. Primary antibodies were incubated as follows: GAPDH (1:10,000, Abcam, USA), LOXL1 (1:1000, Bosterbio, China), INOS (1:1000, Bosterbio, China), COX2 (1:1000, Proteintech, China), IL-6 (1:1000, Affinity, China), AKT (1:1000, OriGene, China), p-AKT (1:1000, OriGene, China), PI3K (1:1000, CST, USA), p-PI3K (1:1000, CST, USA).

### Small interfering RNA transfection

Si-LOXL1 was purchased from GenePharma Biotech (Shanghai, China). Human synovial sarcoma cells SW982 cells were inoculated in 6-well plates and left to transfect after 24 h. The synovial cells were treated with LOXL1-SiRNA1, LOXL1-SiRNA2, LOXL1-SiRNA3 and NC-SiRNA, respectively. The Si-RNAs were introduced into synovial cells with Lipo3000 transfection test (Invitrogen, USA). After 24 h, synovial cells were stimulated with TNF-α (20 ng/ml) for a period of time for further analysis. Real-time fluorescence quantitative polymerase chain reaction and western blotting were used to detect transfection efficiency. The siRNA sequences are provided in Supplementary Table 2.

### Histological processing and immunohistochemistry

Clinical specimens, tissue specimens were fixed in 4% paraformaldehyde and embedded in paraffin. Synovial samples were cut into 4 μm thick slices and analyzed for tissue specimens using hematoxylin-eosin (H&E) staining. Immunohistochemical methods were used to detect the expression of LOXL1 and the corresponding inflammatory factors in human synovial membranes. Primary antibodies (anti-LOXL1, anti-IL-6, anti-COX2, anti-INOS) were incubated on tissue sections overnight at 4°C, then incubated with biotin-labeled secondary antibodies, followed by DAB staining and hematoxylin staining of nuclei. Finally the sections were dehydrated, washed, and sealed. The sections were observed under a Leica light microscope (Wetzlar, Germany) to visualize the immunostaining. Positive cells appeared brownish or yellowish, while negative cells did not stain or stained very weakly.

### Immunofluorescence staining

SW982 cells were inoculated into a 24-well plate with circular slides and allowed to fully adhere to the wall. Cells were treated with TNF-α (20 ng/ml) for 48 hours. After treatment, cells were fixed with 4% paraformaldehyde for 20 min and then permeabilized with 0.5% Triton X-100 for 15 min. They were co-incubated with bovine serum albumin for 30 min at room temperature. Subsequently, the cells were incubated with anti-IL-6/COX2 antibody (1:200) overnight at 4°C. The next day, the corresponding fluorescent secondary antibodies were incubated for 2 hours at room temperature. After the addition of the fluorescent secondary antibody, subsequent manipulations were handled in the dark. Next, the nuclei were stained with 4,6-diamidino-2-phenylindole (DAPI) for 10 min. The cells were kept in a moist environment throughout the procedure. Finally, circular slides with cells were fixed on slides and cells were observed using a high-resolution laser confocal scanning microscope.

### Microarray data

We developed a diagnostic model for rheumatoid arthritis and obtained gene expression profile data for GSE12021, GSE55235 and GSE55457 from GEO (https://www.ncbi.nlm.nih.gov/geo/). The three datasets included 29 normal samples and 65 patient samples. The population demographic information of the three data sets is shown in Table 2. Because they share the same platform, all three datasets can be combined into one metadata cohort for comprehensive analysis. In addition, we were able to also implement batch effect removal using the SVA package of the R program.

**Table 2:** The information of those three datasets involving patients from the GEO database.

**GSE number**	**Platform**	**Samples**		**Average age**		**Gender**		**Gene**
		**Controls**	**Patients**	**Controls**	**Patients**	**Female**	**Male**	
GSE55235	GPL96	10	20	/	/	/	/	mRNA
GSE55457	GPL96	10	23	51	65.39	20	13	mRNA
GSE12021	GPL96	9	22	50.22	68.05	19	12	mRNA

### Analysis of basement membrane-associated differential genes and integrated microarray datasets

Three data sets were merged into one metadata cohort, and batch effects were processed and eliminated. The metadata cohort was first intersected with 224 basement membrane-associated genes to obtain the set of basement membrane-associated gene expressions in normal samples and samples from rheumatoid arthritis patients in the metadata cohort. Basement membrane-associated differential genes between rheumatoid arthritis and healthy samples were identified using the Limma package in R. |Log2FC|>1, *P* < 0.05, and false discovery rate (pseudo-discovery rate) <0.05 were used as thresholds for screening differentially expressed genes. 224 basement membrane gene sets will be displayed in Supplementary Table 3.

### Functional enrichment analysis

The Gene Ontology (GO) and Kyoto Encyclopedia of Genes and Genomes (KEGG) pathways were used to analyze patients between high-risk and low-risk groups through the “clusterProfiler” R package [20]. *P*-values < 0.05 were considered statistically significant for the results of GO and KEGG pathway analysis. Disease Ontology (DO) enrichment analysis was performed using the clusterProfiler package in R and the DOSE package for differentially expressed genes.

### Diagnostic gene selection

Two machine learning algorithms were used to predict diagnostic factors that may be associated with rheumatoid arthritis. LASSO is a regression analysis algorithm that uses regularization to improve prediction accuracy by identifying genes with significant differences between basement membrane-associated RA and normal subjects through the “glmnet” package in R. Support Vector Machines (SVMs) are widely used for classification and regression analysis. We used recursive feature elimination (RFE) to circumvent overfitting. To obtain the most significant gene sets, SVM Recursive Feature Elimination (SVM-RFE) was used to screen appropriate features.

### CiberSort analysis

CIBERSORT (http://cibersort.stanford.edu/) is a deconvolution algorithm based on a linear regression model that can characterize the cellular composition of complex tissues from their gene expression profiles [21]. We determined the immune response of 22 immune cells using the CIBERSORT calculator and analyzed the association of these immune cells with the expression of key genes in normal and RA samples to determine the role and association of these immune cells in disease development.

### Statistical analysis

We used *t*-tests to compare genetic differences between patients with rheumatoid arthritis and healthy individuals. ROC curves and AUC values were calculated by R package “pROC” to verify the effect of key genes on the classification between rheumatoid arthritis patient specimens and healthy specimens. Statistical analysis was performed using R program 3.5.3 and Prism (GraphPad Prism, USA), with statistically significant differences when ^*^p < 0.05, ^**^p < 0.01, ^***^p < 0.001 and ^****^p < 0.0001.

### Data availability statement

The raw data supporting the conclusions of this article will be made available by the authors, without undue reservation.

## RESULTS

### Determination of differentially expressed genes localized to basement membrane in rheumatoid arthritis

**Figure 1:**
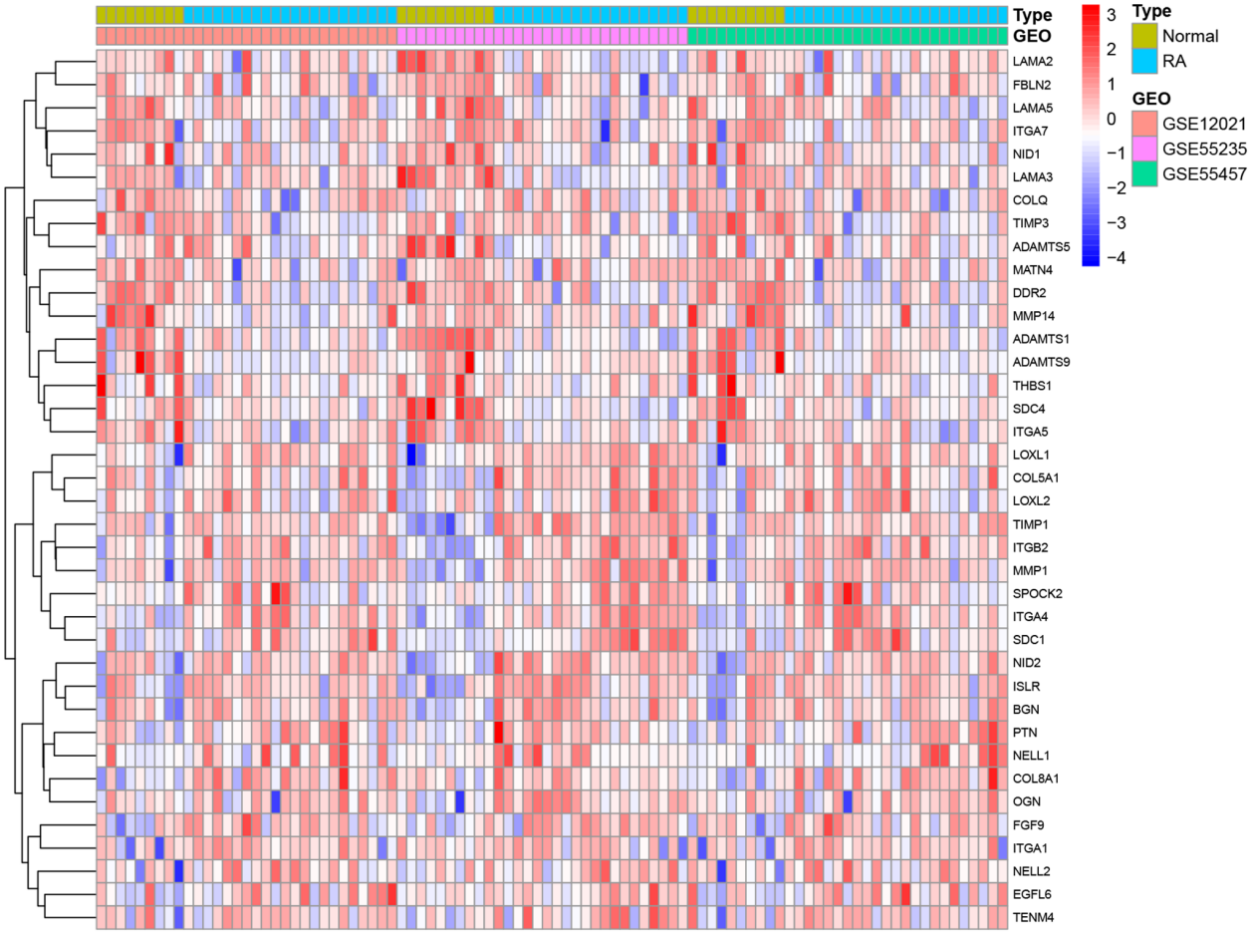
Differences between rheumatoid arthritis and healthy specimens.

In this study, 224 genes localized to the basement membrane and data from a total of 29 normal and 65 patient samples from three GEO datasets (GSE12021, GSE55235, and GSE55457) were examined. The three data sets were merged into a metadata cohort and their intersection with basement membrane-associated genes was derived. The differentially expressed genes from the raw data were investigated by the LIMMA package. In total, we identified 38 differentially expressed genes associated with basement membrane: 21 genes were significantly upregulated and 17 genes were significantly downregulated (Figure 1).

### Functional enrichment analysis

**Figure 2:**
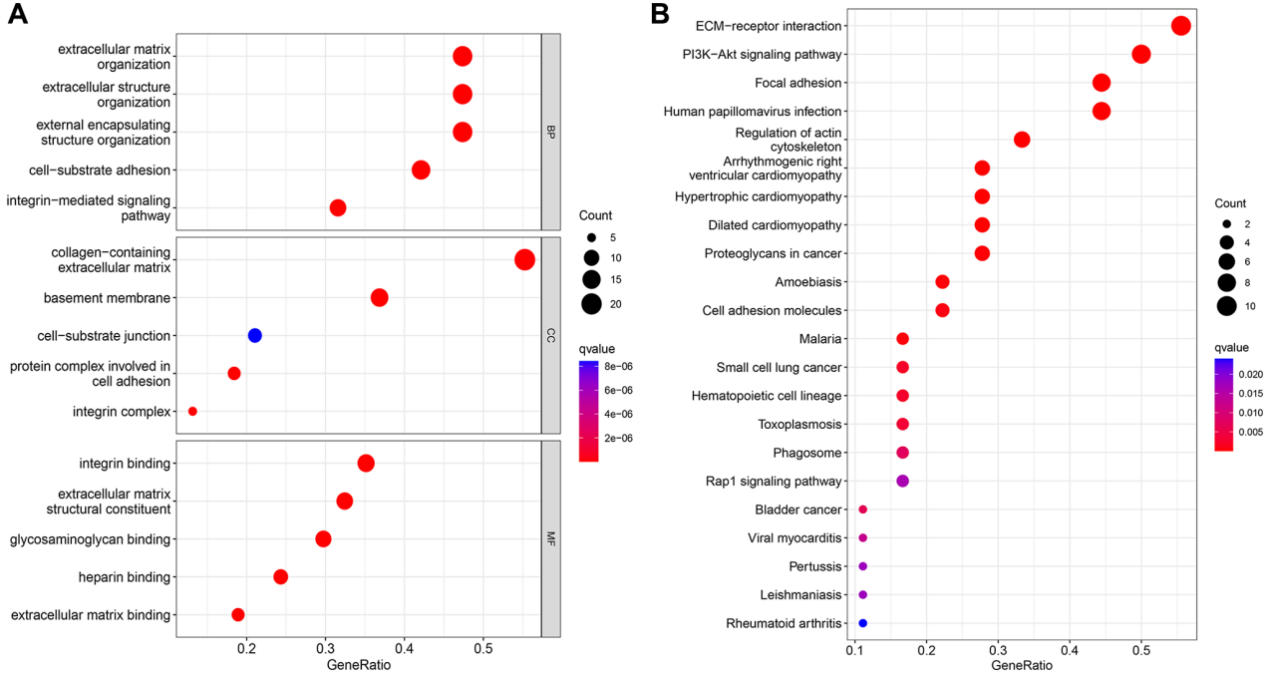
GO analysis (**A**) and KEGG analysis (**B**) of 39 differentially expressed genes.

GO and KEGG analysis were conducted on 38 basement membrane-associated differentially expressed genes using the ClusterProfile R software package to explore their biological functions in rheumatoid arthritis. The results showed that 38 basement membrane-associated differentially expressed genes were mainly involved in extracellular matrix organization, extracellular structural organization, external encapsulated structural organization, cell-substrate adhesion, collagen-containing extracellular matrix, basement membrane, integrin binding, extracellular matrix structural constituent and glycosaminoglycan binding. Meanwhile, KEGG analysis showed significant enrichment in signaling pathways, including PI3K/Akt signaling pathway, ECM-receptor interaction, Focal adhesion, Human papillomavirus infection and Regulation of the actin cytoskeleton (Figure 2).

### Identification of diagnostic markers and analysis of their diagnostic value

**Figure 3:**
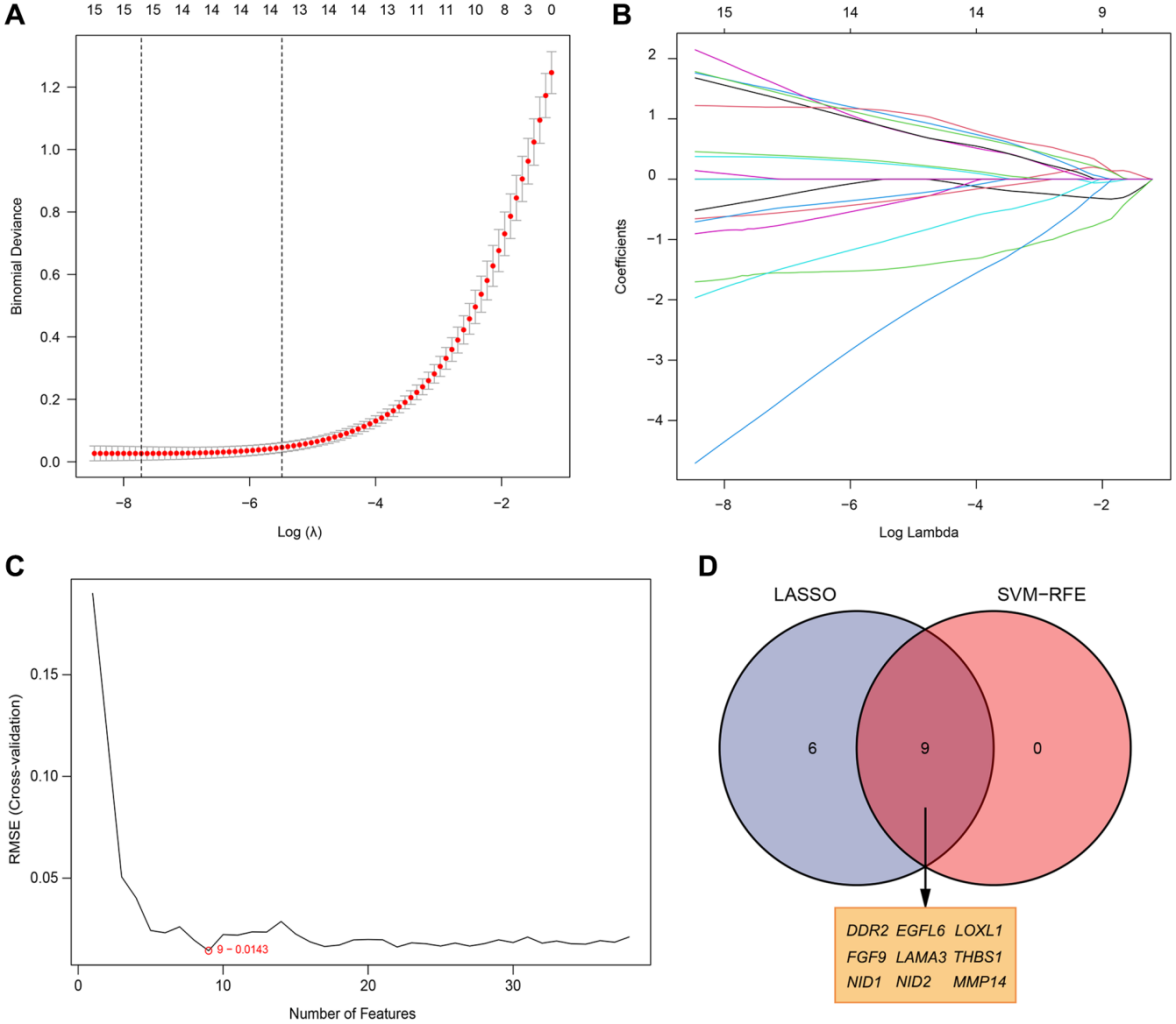
**Selection of diagnosis marker candidates for RA.** (**A**, **B**) LASSO regression model; (**C**) A plot of biological marker screening via the SVM-RFE arithmetic; (**D**) Venn graph displaying 9 diagnosis biomarkers shared by LASSO and SVM-RFE.

**Figure 4:**
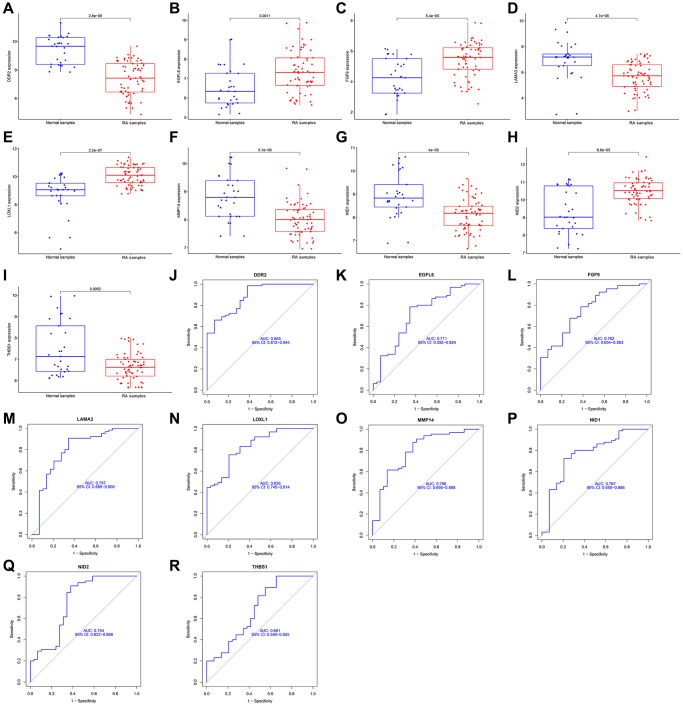
**Expression and diagnostic significance of 9 basement membrane related genes in RA.** (**A**–**I**) DDR2, LAMA3, MMP14, NID1 and THBS1 expression was distinctly downregulated in RA samples; LOXL1, EGFL6, FGF9 and NID2 expression was distinctly upregulated in RA samples. (**J**–**R**) ROC assays for 9 basement membrane related genes.

Two different algorithms were used to predict potential biomarkers, and a lasso regression algorithm was used to identify differences in gene expression localized to the basement membrane between healthy and rheumatoid arthritis specimens, resulting in 15 variables as diagnostic markers for RA. (Figure 3A, 3B) A subset of 9 features in differentially expressed genes was analyzed by SVM-RFE (Figure 3C). Finally, feature genes that overlapped between these two algorithms were obtained: DDR2, EGFL6, LOXL1, FGF9, LAMA3, THBS1, NID1, NID2 and MMP14, which are localized on the basement membrane and may be involved in the development of rheumatoid arthritis (Figure 3D). Their three data sets (GSE12021, GSE55235, and GSE55457) were merged for analysis. Compared with healthy samples, DDR2, LAMA3, MMP14, NID1 and THBS1 expression levels were significantly down-regulated in RA samples, while LOXL1, EGFL6, FGF9 and NID2 expression was significantly up-regulated. (Figure 4A–4I). We performed ROC analysis on the diagnostic value of the overlapping nine differentially expressed genes for rheumatoid arthritis (Figure 4J–4R). Area under the ROC curve (AUC) value greater than 0.8 was considered as a condition for good diagnostic value, and DDR2 and LOXL1 performed well in distinguishing normal and rheumatoid arthritis samples. The AUC value for DDR2 was 0.885, and the ROC value for LOXL1 was 0.836.

### Relationship between basement membrane associated genes and immune cells

**Figure 5:**
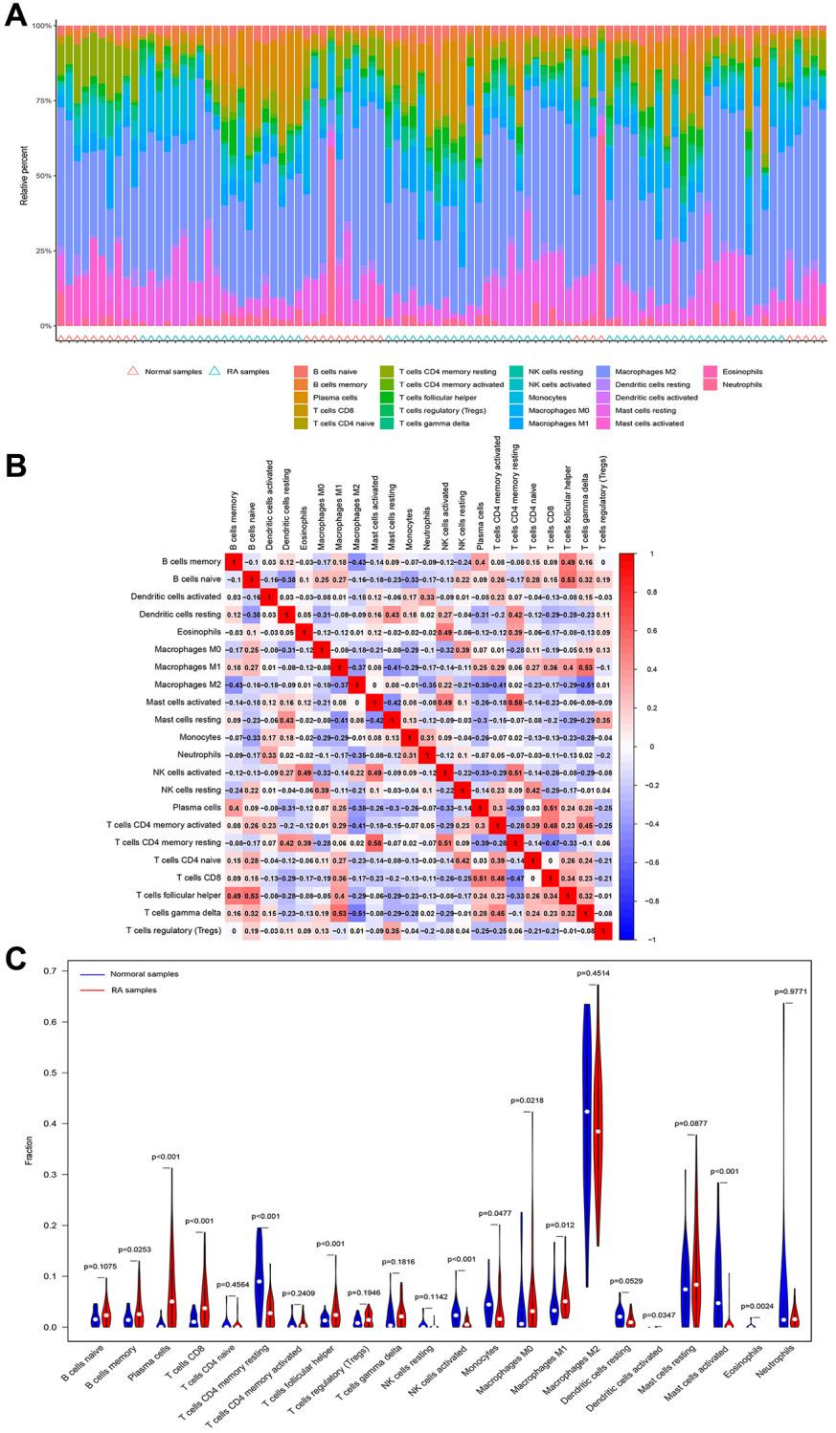
**Immune infiltration status.** (**A**, **B**) The percentage of the 22 immunocytes identified via the CIBERSORT arithmetic. (**C**) The diversities in the architecture of immunocytes between healthy and RA specimens.

**Figure 6:**
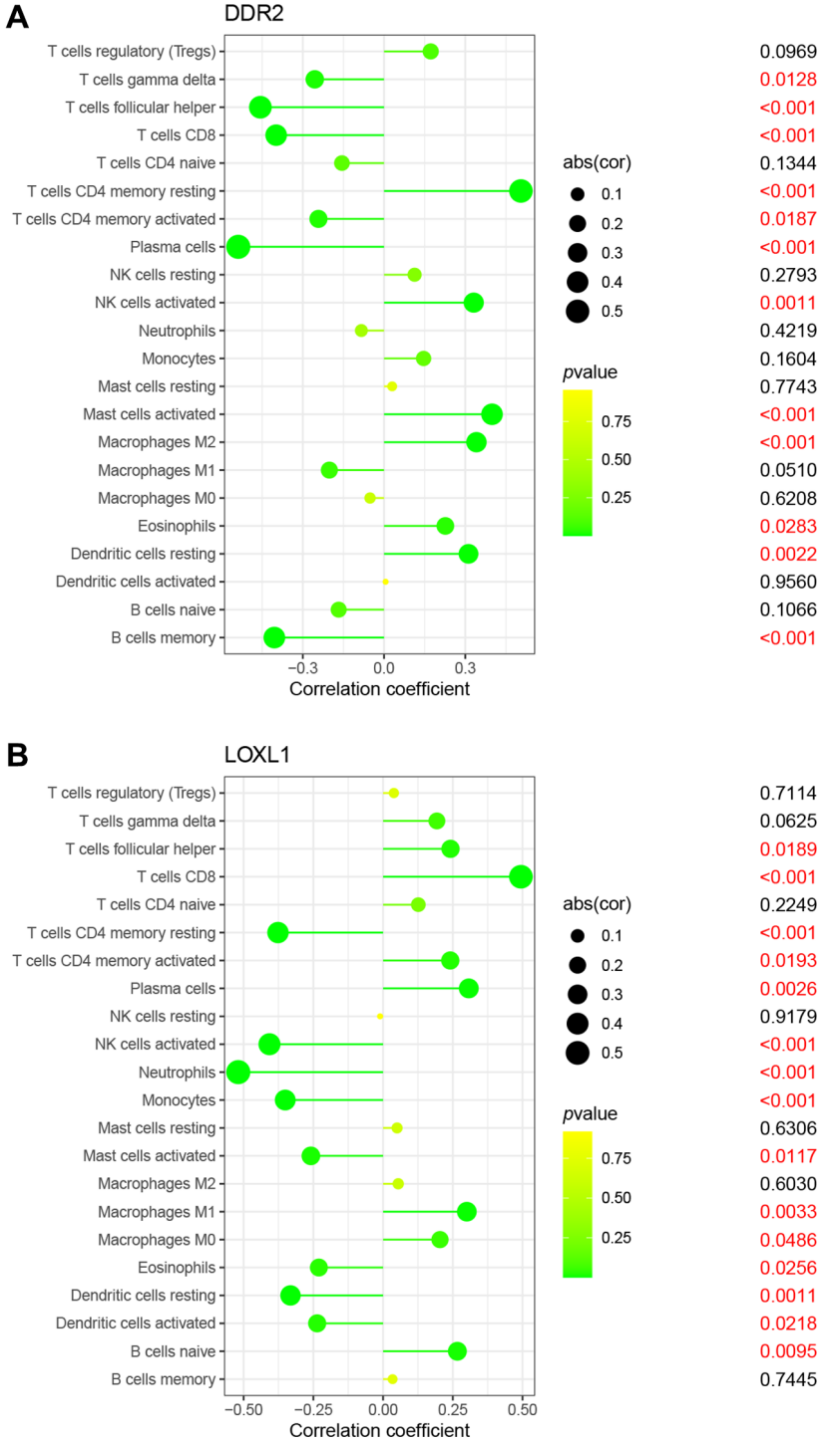
Correlation between DDR2 (**A**), LOXL1 (**B**), and infiltrating immune cells in RA and normal samples.

It is well known that rheumatoid arthritis is a typical systemic disease caused by autoimmune factors. Therefore, we investigated the ratio of LOXL1 and DDR2 to different immune cell infiltrations between rheumatoid arthritis and normal samples, aiming to determine the association between the infiltration status of immune cells and the expression levels of LOXL1 and DDR2. Figure 5A, 5B shows the characteristics of 22 kinds of immune cells through CIBERSORT approach, as well as its composition in rheumatoid arthritis specimens in relation to normal immune cells. We also investigated the differences between immune cellular constructs between rheumatoid arthritis and normal samples. The results showed abnormal regulatory levels of B cells memory, plasma cells, T cells CD8, T cells CD4 memory resting, T cells follicular helper, NK cells activated, Monocytes, Macrophages M0 and M1, Dendritic cells activated, Mast cells activated and Eosinophils in normal samples and rheumatoid arthritis samples (Figure 5C). In addition, we further explored the association of DDR2 and LOXL1 expression with the degree of immune infiltration, and found that DDR2 was associated with T cells CD8, T cells follicular helper and plasma cells, while LOXL1 was associated with T cells CD4 memory resting, NK cells activated, and Neutrophils (Figure 6). Our findings suggested that DDR2 and LOXL1 may be involved in the progression of rheumatoid arthritis by influencing the activities of multiple immune cells.

### Upregulation of LOXL1 expression in human rheumatoid arthritis synovium

**Figure 7:**
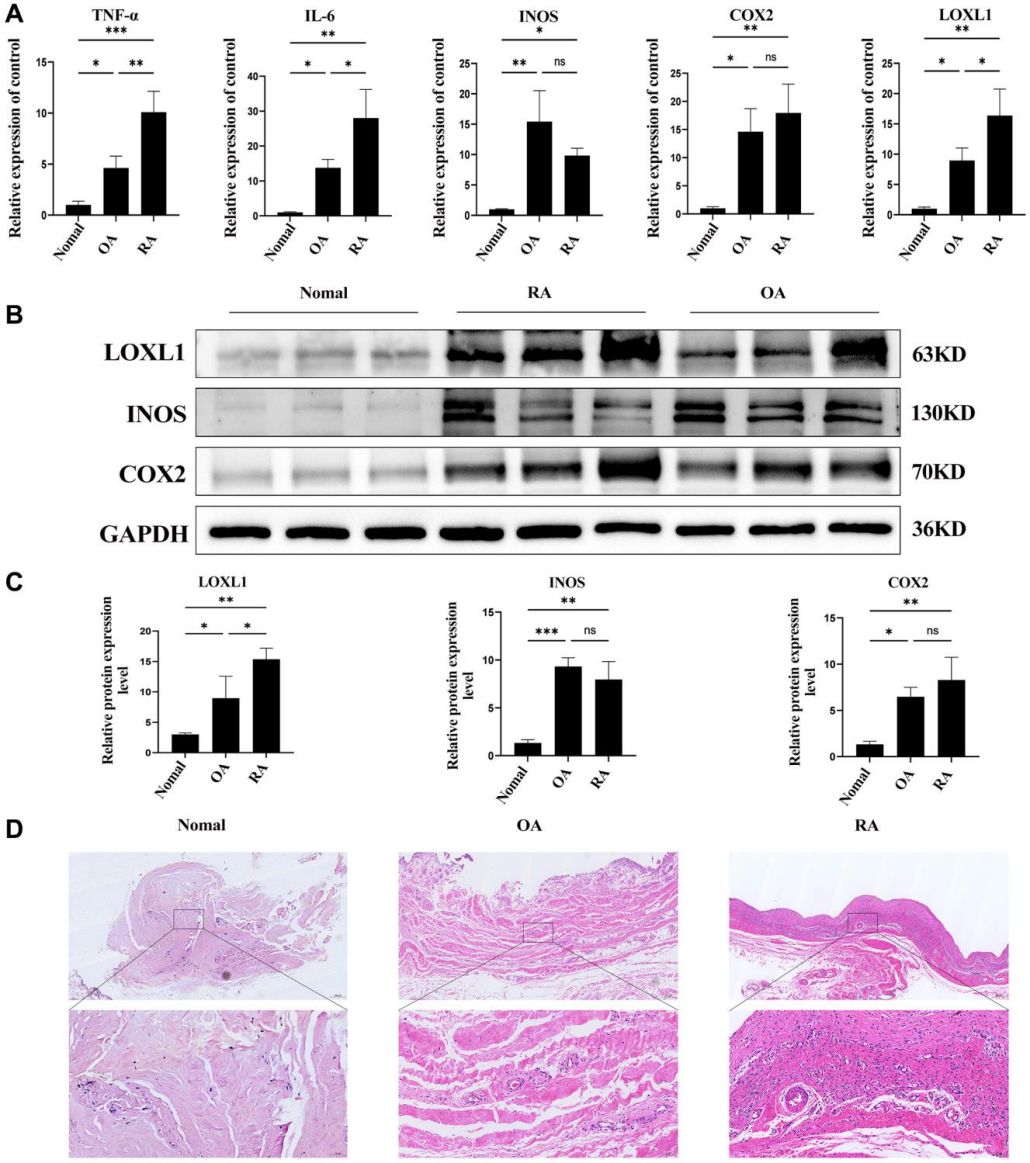
**LOXL1 expression in human RA synovial membrane.** (**A**) Expression of LOXL1, TNF-α, IL-6, INOS and COX2 was detected in RA synovium, OA synovium and normal synovium by qPCR. (**B**) LOXL1, INOS and COX2 protein expression level was assessed in RA synovium, OA synovium and normal synovium using western blot. (**C**) Quantitative analysis of western blot results. (**D**) HE staining of RA synovium, OA synovium and normal synovium. Low magnification scale bar = 200 μm and High magnification scale bar = 50 μm. ^*^P < 0.05, ^**^P < 0.01, ^***^P < 0.001.

**Figure 8:**
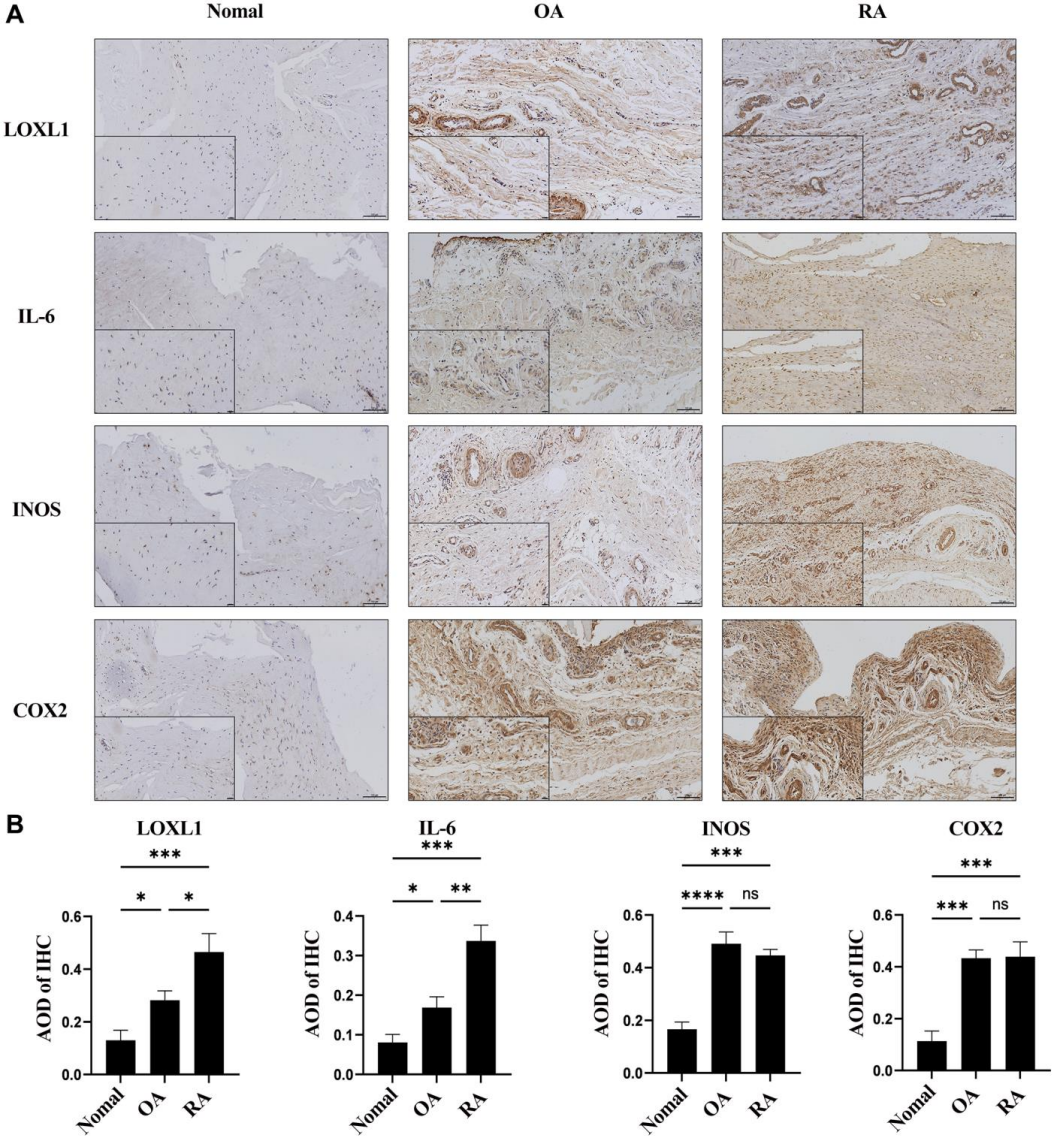
LOXL1 expression was measured in RA synovium, OA synovium, and normal synovium through immunohistochemistry (**A**) and its quantitative analysis (**B**). Low magnification scale bar = 100 μm and High magnification scale bar = 20 μm. ^*^P < 0.05, ^**^P < 0.01, ^***^P < 0.001, ^***^P < 0.0001.

In chronic osteoarthritis, there is a strong association between discoid peptide receptor 2 (DDR2) and matrix metalloproteinases (MMPs). A large number of studies have demonstrated the involvement of DDR2 in the disease process of rheumatoid arthritis and osteoarthritis [22–24]. Focusing on LOXL1, which has been little explored in chronic osteoarthritic diseases including rheumatoid arthritis and osteoarthritis, we examined the expression levels of LOXL1 in synovial tissues of patients with chronic osteoarthritis and normal humans using RT-qPCR and western blot methods. The results showed that the expression of LOXL1 in synovial tissue of patients was significantly higher than that in normal synovial tissue (Figure 7A, 7B). Quantitative analysis confirmed the observations of western blot (Figure 7C). Through HE staining, we observed that rheumatoid arthritis synovial cells proliferated in a papillary or villous pattern compared with the synovium of normal and osteoarthritic patients, with a large infiltration of immune cells (Figure 7D). Meanwhile, the expression of LOXL1 and inflammatory factors such as IL-6, INOS and COX2 in synovial tissues was detected by immunohistochemistry. We obtained the same results as previous experiments (Figure 8A), which was confirmed by the quantitative analysis of the above results (Figure 8B). The above data show a significant upregulation of LOXL1 expression in chronic bone and joint diseases, particularly in rheumatoid arthritis, suggesting a possible role of LOXL1 in the development of rheumatoid arthritis. Later we will focus on exploring the role of LOXL1 in the development of rheumatoid arthritis.

### Elevated LOXL1 expression in TNF-α-stimulated human osteosarcoma synovial cells

**Figure 9:**
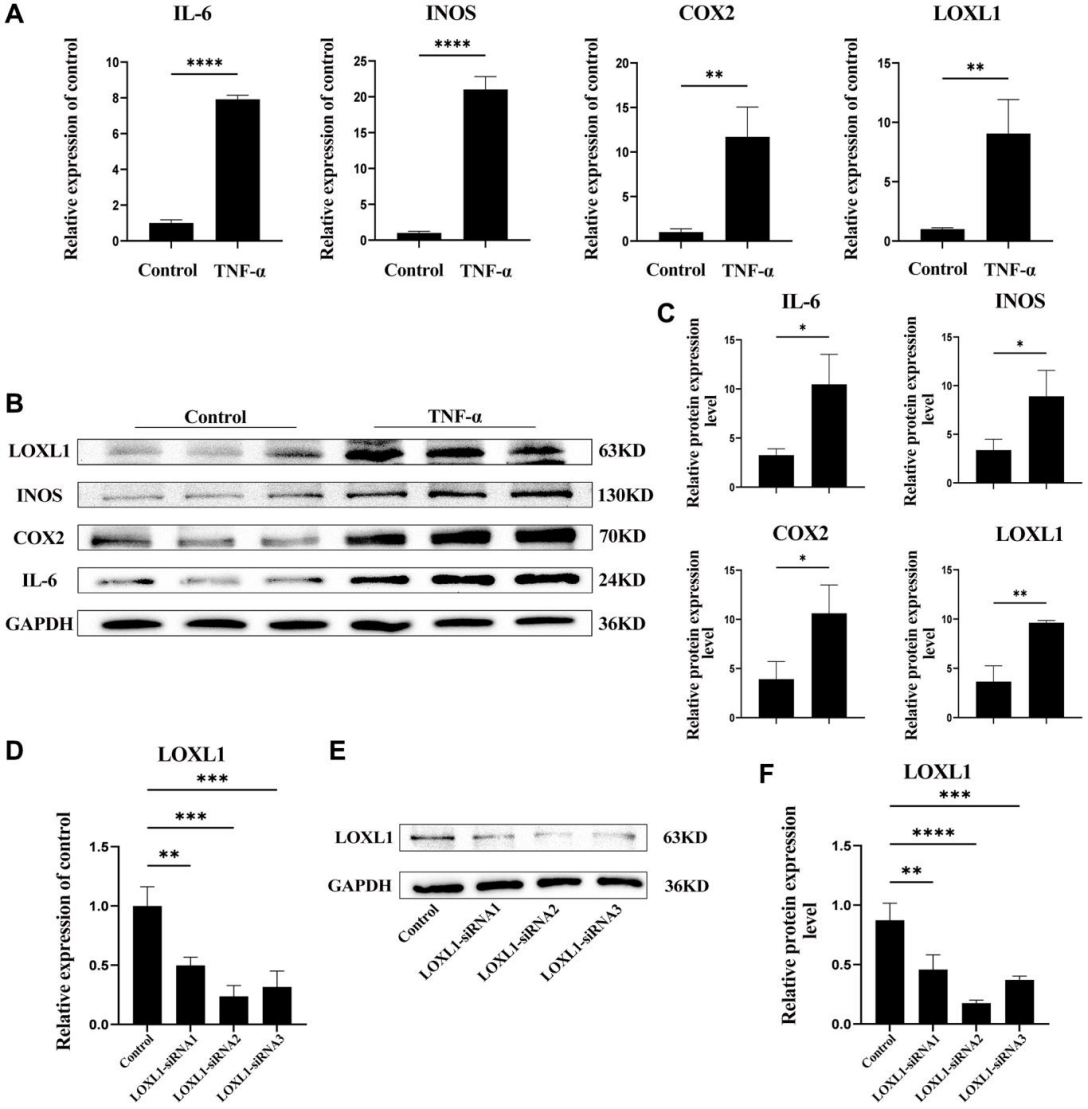
**LOXL1 expression in TNF-α-stimulated SW982 cells.** (**A**) LOXL1, IL-6, INOS and COX2 mRNA expression levels were analyzed using qPCR. (**B**) SW982 cells were cultured and stimulated with TNF-α (20ng/mL). LOXL1, IL-6, INOS and COX2 protein levels were detected using western blot at the indicated time. (**C**) Quantitative analysis of western blot results. (**D**, **E**) qPCR and western blot were used to verify the efficiency of downregulation of LOXL1 gene expression by small interfering RNA. (**F**) Quantitative analysis of western blot results. ^*^P < 0.05, ^**^P < 0.01, ^***^P < 0.001, ^***^P < 0.0001.

To further verify the expression of LOXL1 in rheumatoid arthritis synovium, we used TNF-α to stimulate human osteosarcoma synovial cells for 48 h, extracted total protein and total mRNA, and detected the expression of LOXL1, INOS, COX2, and IL-6 in them through RT-qPCR and western blot. After TNF-α stimulation, the RT-qPCR results showed that the mRNA expression of LOXL1, INOS, COX2 and IL-6 were significantly increased compared with the untreated group (Figure 9A). Meanwhile, results of the western blot showed that the expression of LOXL1, INOS, COX2 and IL-6 were also significantly upregulated (Figure 9B), which was also confirmed by the results of quantitative analysis (Figure 9C).

### Downregulation of LOXL1 expression may suppress RA synovial inflammation by affecting the PI3K/ AKT pathway

**Figure 10:**
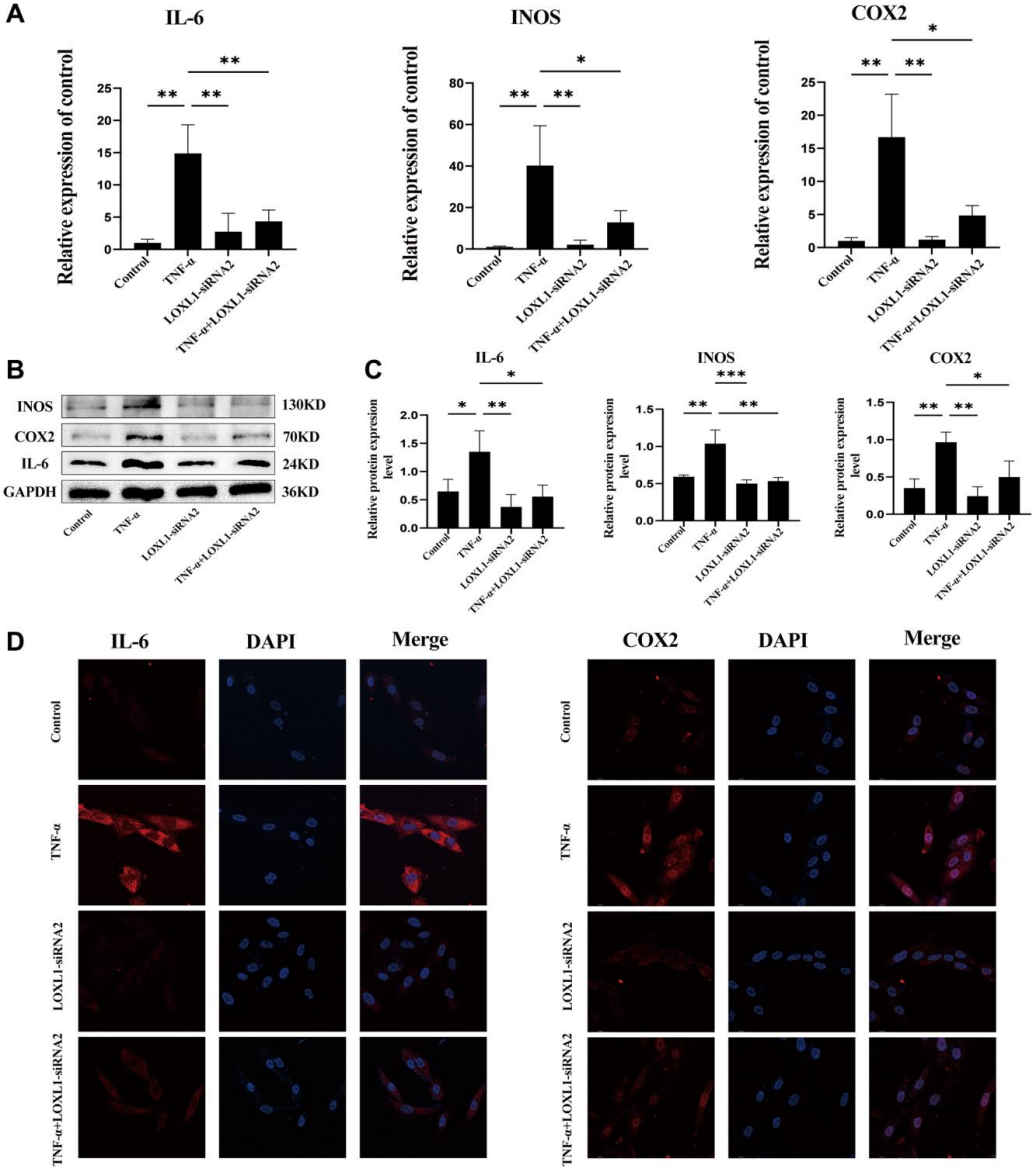
**Knockdown of LOXL1 inhibited inflammatory reaction of RA synovial cells.** (**A**) IL-6, INOS and COX2 mRNA expression levels were analyzed using qPCR. (**B**) IL-6, INOS and COX2 protein levels were detected using western blot. (**C**) Quantitative analysis of western blot results. (**D**) Validation of IL-6 and COX2 expression using cellular immunofluorescence. ^*^P < 0.05, ^**^P < 0.01, ^***^P < 0.001.

**Figure 11:**
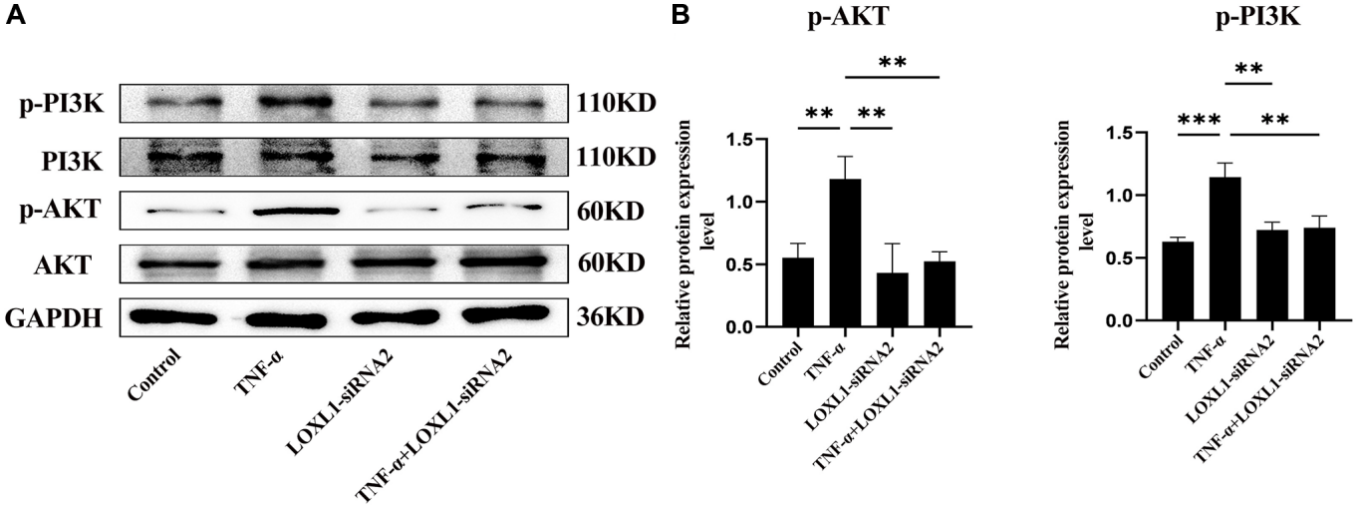
**Knockdown of LOXL1 may inhibit the inflammatory response in RA synoviocytes by suppressing the PI3K/AKT cell signaling pathway.** (**A**) p-PI3K, PI3K, p-AKT and AKT protein levels was detected using western blot. (**B**) Quantitative analysis of western blot results. ^*^P < 0.05, ^**^P < 0.01, ^***^P < 0.001.

We used small interfering RNA (SiRNA) to down-regulate LOXL1 expression, RT-QPCR (Figure 9D) and western blot (Figure 9E, 9F) to detect the silencing efficiency. Based on the knockdown efficiency, we employed LOXL1-siRNA2 to silence the expression of LOXL1. Human synoviocytes were treated with LOXL1-siRNA2 using TNF-α stimulation for 48 h. RT-qPCR and western blot results showed that the expression of INOS, COX2 and IL-6 was significantly increased after TNF-α stimulation and significantly suppressed after silencing LOXL1 (Figure 10A, 10B). These observations were confirmed by quantitative analysis (Figure 10C). Meanwhile, through immunofluorescence, we verified that the fluorescence intensity and density of IL-6 and COX2 were significantly downregulated when LOXL1 expression was down-regulated compared with the TNF-α treated group (Figure 10D). To further investigate the role of LOXL1 in the development of rheumatoid arthritis, total protein was extracted by adding TNF-α stimulation for 30 min after downregulation of LOXL1 expression. We found that the phosphorylation levels of PI3K and AKT were significantly decreased after LOXL1 knockdown compared with the TNF-α stimulated group (Figure 11A), which was also confirmed by quantitative analysis (Figure 11B). In summary, basement membrane-localized LOXL1 may mediate the inflammatory response of rheumatoid arthritis synovial cells by influencing the activation of the PI3K/AKT pathway, thereby promoting their disease progression.

## DISCUSSION

There are no definitive diagnostic criteria for rheumatoid arthritis, and patients typically present with joint tenderness and swelling, morning stiffness, and abnormalities on laboratory tests such as elevated C-reactive protein or erythrocyte sedimentation rate. Unfortunately, these symptoms are not specific for the diagnosis of rheumatoid arthritis, which can be present in many joint diseases, such as reactive arthritis, psoriatic arthritis, osteoarthritis, infectious arthritis, or some rarer autoimmune diseases such as connective tissue diseases (e.g., rash, oral ulcers, alopecia, Raynaud’s phenomenon, etc.) [25]. Early diagnosis and treatment can prevent or retard the progression of joint damage in up to 90% of patients with rheumatoid arthritis [26]. The basement membrane, a specific extracellular matrix manifestation, is not only involved in tissue composition, but also mediates cellular behavior as an important regulator [27]. In recent years, the possible mechanisms of basement membrane in the progression of diseases involved in Alport syndrome, Goodpasture syndrome, diabetes mellitus and cancer have been widely studied [27, 28]. Although numerous studies have shown the important role of basement membranes in most human diseases, especially in autoimmune diseases, the role of their associated genes in the classic autoimmune disease rheumatoid arthritis has not yet been elucidated [29, 30]. In this study, we synthetically analyzed a gene set containing 224 genes localized to the basement membrane and three GEO datasets to obtain 38 basement membrane-associated differentially expressed genes in RA samples and healthy samples. GO analysis showed that 38 differentially expressed genes were mainly involved in extracellular matrix organization, basement membrane genes, integrin binding and other biological functions. Referring to previous studies, we obtained the signaling pathways enriched by these 38 differentially expressed genes through KEGG analysis, such as the interaction between PI3K/Akt signal pathway and extracellular matrix receptors [31]. These findings indicated that basement membrane related genes are actively involved in the disease process of RA, exerting an important role.

To obtain biomarkers with diagnostic value, we applied two machine learning algorithms to analyze 38 basement membrane-associated differentially expressed genes, and identified 9 genes with diagnostic value. Finally two important genes were obtained, namely, DDR2 and LOXL1. Meanwhile, the imbalance of the immune system and changes in the composition of immune cells play an important role in the development of specific diseases [32]. It is necessary to explore immune cell infiltration and its components from the perspective of the immune system since rheumatoid arthritis is a typical autoimmune disease, so as to reveal the causal relationship at the RA molecular level and design new immunotherapy targets. We found that DDR2 was associated with immune cells such as CD8-positive T cells, follicular helper T cells, and plasma cells, while LOXL1 was associated with resting CD4-positive T cells, activated NK cells, and neutrophils. DDR2 is a tyrosine kinase with fibrillar collagen as ligand, which regulates cell proliferation and matrix metalloproteinase expression by binding to collagen [33, 34]. A large number of studies have suggested the involvement of DDR2 in the disease process of chronic osteoarthritis [35, 36]. We focused on the more interesting LOXL1, which belongs to the amine oxidase subfamily and contains tropinone - a modified tyrosine side chain that is used as a redox factor [37]. The LOX family is expressed in adult organs such as the heart, lung, placenta and kidney, and LOXL1 gene expression is particularly high in the heart, placenta and skeletal muscle [38, 39]. The downregulation of LOXL1 expression resulted in the inability of mice to synthesize elastic fibers normally after childbirth, leading to postnatal pelvic organ prolapse and concomitant pro-elastin accumulation [40]. However, the expression and role of LOXL1 in rheumatoid arthritis remain unclear. Here, our team reported for the first time that the basement membrane-associated gene LOXL1 was significantly upregulated in RA samples compared with healthy samples, suggesting that LOXL1 may be involved in disease progression in rheumatoid arthritis.

Normal synovium consists of fibroblast-like synoviocytes and macrophages [40]. Synovial inflammation plays a central role in the development of rheumatoid arthritis, and its inflammatory milieu is regulated by a complex network of cytokines and chemokines, including tumor necrosis factor, interleukin 6, and possibly granulocyte colony-stimulating factor [41]. Early cartilage damage occurs in rheumatoid arthritis in the context of immune activation, serving as a key trigger for synovial cell responses. The molecular steps of cartilage matrix degradation in chronic inflammatory joint disease overlap with those in degenerative joint disease [42]. We first investigated LOXL1 expression in normal subjects, degenerative arthritis and rheumatoid arthritis. Real-time PCR, western blot and immunohistochemistry demonstrated that LOXL1 expression was significantly upregulated in synovial tissues of common osteoarthritic diseases, especially in rheumatoid arthritis. To further validate the role of LOXL1 in rheumatoid arthritis, we simulated the inflammatory environment of rheumatoid arthritis *in vitro* by stimulating SW982 cells with TNF-α [43]. RT-qPCR results showed that the expression of IL-6, INOS, COX2 and LOXL1 was upregulated after TNF-α action, and the same results were obtained at the protein level. Next, we sought to elucidate the role of LOXL1 in the development of RA by inhibiting its expression in synovial cells using small interfering RNA. Through immunofluorescence technique, we observed that the fluorescence intensity and density of IL-6 and COX2 were significantly downregulated after downregulation of LOXL1 expression compared with the TNF-α-treated group alone. The same results were verified at both the mRNA and protein levels. Therefore, we concluded that LOXL1 was involved in the inflammatory process of RA synoviocytes. Finally, we tried to explore the possible mechanisms of LOXL1 involvement in the development of RA synovial inflammation. The results of KEGG enrichment analysis showed that the presence of PI3K/AKT signaling pathway in the disease process. The PI3K/AKT signaling pathway is involved in a wide variety of biological behaviors in the human body, and more than 150 proteins have been identified as effectors of PI3K/AKT mediating cell cycle, cell survival, inflammation, and apoptosis [44, 45]. Aberrant regulation of PI3K/AKT signaling pathway not only leads to overexpression of inflammatory factors, but also affects osteoclast differentiation and production. Additionally, it is considered as a potent target for the treatment of rheumatoid arthritis. *In vivo* and *in vitro* studies have shown that PI3K inhibition by ZSTK474 may inhibit synovial inflammation and cartilage destruction in RA patients [46, 47]. We concluded from the study of basement membrane-associated gene LOXL1 that LOXL1 may decelerate RA synovial inflammation by inhibiting the activation of PI3K/AKT signaling pathway, and LOXL1 may be a potential diagnostic biomarker and therapeutic target for rheumatoid arthritis.

The present study also has some limitations. First, the clinical specimens obtained were limited. The number of clinical specimens should be expanded. Second, our work can only show the association between basement membrane-associated genes and immune cells in the development of RA, but cannot prove the causal relationship between the two. Finally, we have not been able to clarify how LOXL1 plays a role in the development of rheumatoid arthritis *in vivo*. These three directions will be the focus of our future work.

## Supplementary Materials

Supplementary Tables 1 and 2

Supplementary Table 3
