# Supplementary material for: Mechanism of lysine oxidase-like 1 promoting synovial inflammation mediating rheumatoid arthritis development
Source: Aging (Albany NY). 2024 Jan 12;16(1):928–47. doi: 10.18632/aging.205429 (PMC10817408; doi:10.18632/aging.205429)
Supplement: Supplementary Tables 1 and 2 [file aging-16-205429-s001.pdf]

SUPPLEMENTARY TABLES

Supplementary Table 1. Primer sequences for GAPDH, INOS, COX2, TGFβ, IL6 and LOXL1.

Primer name	Primer sequence
H-GAPDH-F	GGAAGCTTGTCATCAATGGAAATC
H-GAPDH-R	TGATGACCCTTTTGGCTCCC
H-INOS-F	GACTCACAGCCTTTGGACCTCA
H-INOS-R	GCTGGATGTCGGACTTTGTAGATT
H-COX2-F	AAGACAGATCATAAGCGAGGGC
H-COX2-R	AAACCGTAGATGCTCAGGGACT
H-TGFβ-F	CAGCAACAATTCCTGGCGATA
H-TGFβ-R	GCTAAGGCGAAAGCCCTCAAT
H-IL6-F	ATGAGGAGACTTGCCTGGTGAA
H-IL6-R	CTCTGGCTTGTTCCTCACTACTCTC
H-LOXL1-F	TGCCACCAGCATTACCACAG
H-LOXL1-R	GAGGTTGCCGAAGTCACAGG

Abbreviations: F: Forward; R: Reverse; H: Human.

Supplementary Table 2. Small interfering RNA sequence of LOXL1.

Home-Human	siRNA sequences	
	Sense (5′-3′)	Antisense (5′-3′)
LOXL1-Homo-SiRNA1	AAGCAUCCACUUAUGUGCATT	UGCACAUAAGUGGAUGCUUGC
LOXL1-Homo-SiRNA2	GCUAUGCAUGCACCUCUCATT	UGAGAGGUGCAUGCAUAGCGC
LOXL1-Homo-SiRNA3	ACGUGAACCCAAAGUAUAUTT	AUAUACUUUGGGUUCACGUGC
